# A comparative biomechanical analysis of suprapectineal and infrapectineal fixation on acetabular anterior column fracture by finite element modeling

**DOI:** 10.3906/sag-1806-72

**Published:** 2019-02-11

**Authors:** Mehmet YÜCENS, Kadir Bahadır ALEMDAROĞLU, Ahmet ÖZMERİÇ, Serkan İLTAR, Ahmet Özgür YILDIRIM, Nevres H. AYDOĞAN

**Affiliations:** 1 Department of Orthopedics, Faculty of Medicine, Pamukkale University, Denizli Turkey; 2 Department of Orthopedics, Ankara Training and Research Hospital, Ankara Turkey; 3 Ankara Numune Training and Research Hospital, Ankara Turkey; 4 Department of Orthopedics, Faculty of Medicine, Muğla University, Muğla Turkey

**Keywords:** Acetabular fracture, anterior column, suprapectineal, infrapectineal, fixation, finite element

## Abstract

**Background/aim:**

The aim of this study is to compare the stability and implant stresses of suprapectineal plate with infrapectineal plate in three subconfigurations of the screw types.

**Materials and methods:**

The stabilities of different fixation methods were compared by finite element analysis on six models. Three infrapectineal and three suprapectineal models each with locked, unlocked, or combined screws were employed. Three-dimensional finite element stress analysis was performed by using isotropic materials with a load of 2.3 kN applied at standing positions. Motion at the fracture line was measured on four different points located on the pubic and iliac sides of the fracture line.

**Results:**

Infrapectineal plate fixation with unlocked screws was found to be the most stable fixation method with 0.006 mm displacement of fragments in all axes at standing positions. The suprapectineal unlocked method was found to be the most unstable in standing positions with maximum displacement values of 0.46 mm vertical shear movement in the x-axis, –0.14 mm displacement in the y-axis, and –0.33 mm lateral shear in the z-axis.

**Conclusion:**

The infrapectineal unlocked plate supplies the most stable fixation with the least implant stress, contrary to the suprapectineal unlocked plate, which has the lowest stability and highest implant stresses.

## 1. Introduction

An anterior column fracture of the acetabulum can be fixed by using a suprapectineal plate via an ilioinguinal approach (1,2) or using a infrapectineal plate (3–6) by a modified Stoppa approach. The modified Stoppa approach has the advantage of providing access for an infrapectineal plate, which better supports the quadrilateral surface with a relatively short incision without the need for any major vascular dissections (7,8). Although this approach has been praised for having a relatively less steep learning curve (8) than the suprapectineal approach, it may require more experience to perform since the same critical neurovascular structures lay nearby at risk, in this case undissected and hidden (9–11).

To achieve an anatomical reduction, stable fixation and early rehabilitation are the goals of the treatment for an acetabular fracture (4). A reliable fixation may permit a faster rehabilitation and early ambulation of the patient, which may help to avoid severe complications due to prolonged bed rest (12). Although infrapectineal or suprapectineal plate fixation procedures have been used for fractures of the acetabulum, whether infrapectineal or suprapectineal plate fixation could provide the most stable bone-implant construction in an anterior column fracture of the acetabulum has attracted very little attention in the current literature (13). A recent biomechanical study on transverse fractures reported higher stiffness of two new-generation quadrilateral surface buttressing plates over traditional anterior column plating with a posterior column lag screw and posterior column plating with an anterior column lag screw (5).

The aim of this study is to compare the stability and implant stresses of suprapectineal and infrapectineal plate models in three subconfigurations for anterior column fractures using locked screws only, unlocked screws only, or a combination of both screw types by using finite element modeling. The hypothesis was that suprapectineal and infrapectineal plates would work quite differently under loading conditions due to their specific locations, the former over the acetabular dome and the latter buttressing the quadrilateral surface. Standing positions were tested to mimic the basic physiological loads that patients would experience during the early postoperative period.

## 2. Materials and methods

This investigation was undertaken by Ay Tasarım Ltd. at the Ankara University Faculty of Dentistry. In this study, six different methods that are used for the fixation of anterior column fractures of the acetabulum were compared in terms of stability and strength by utilizing a finite element model. Each of the infrapectineal and suprapectineal approaches were modeled with locked screw fixation, unlocked screw fixation, or a combination of unlocked and locked screw fixation (Figures 1A and 1B). The fracture line was determined as a low anterior column fracture with an associated ischial arm fracture. In models with combined screws, the unlocked screws were fixed earlier than the locked screws. Three-dimensional finite element stress analysis was performed by utilizing isotropic materials. Editing and optimizing, solid meshing, and finite element analysis were performed by a computer with Intel Xeon R CPU 3.30 GHz operator, 500 GB hard disk, 14 GB RAM and Windows 7 Ultimate Version Service Pack 1 operating system, Activity 880 optic scanner (Smart Optics Sensortechnik GmbH, Bochum, Germany), Rhinoceros 4.0 3-D modeling software (McNeel & Associates, Seattle, WA, USA), VRMesh Studio (Virtual Grid Inc., Bellevue City, WA, USA), and Algor Fempro analysis software (ALGOR, Inc., Pittsburgh, PA, USA).

**Figure 1A:**
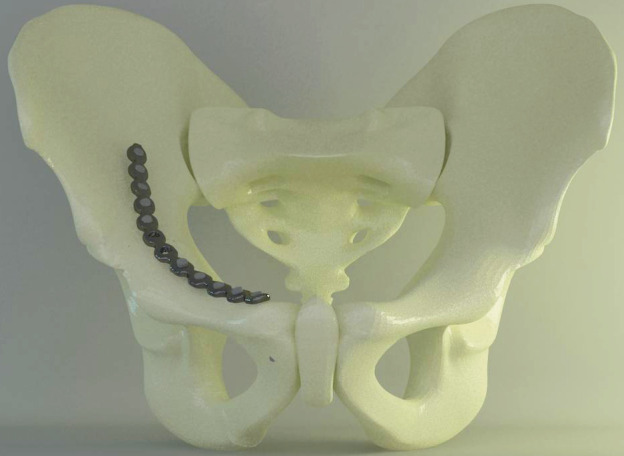
Suprapectineal model with unlocked screws.

**Figure 1B:**
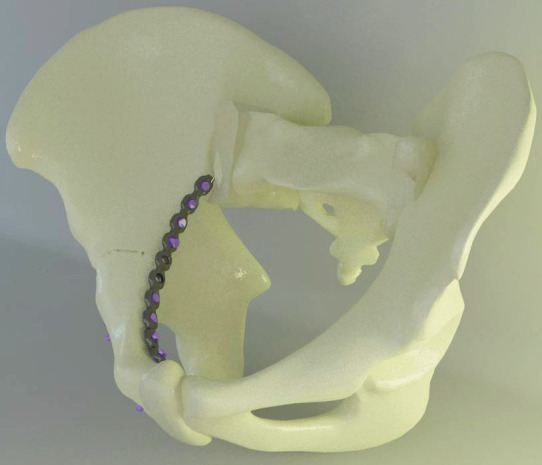
Infrapectineal models with unlocked screws.

A pelvis model was created using data obtained from the study Visible Human Project (http:www.nlm.nih.gov/research/visible/visible human.html). The axial scans that were derived from Visible Human Project were reconstructed in 3D-doctor software by extracting bone tissue. The created 3D model was exported in stl format. In this pelvic model the cortical bone ratio was 28% and spongious bone ratio was 72%. Coefficient of friction was 0.6 in the fracture line. The reconstruction plates had a 3.5 mm thickness and screws had a 4 mm diameter, and were produced by Depuy Synthes (West Chester, PA, USA). Elasticity modulus was assigned as 13.7 GPa for cortical bone, 1 GPa for spongious bone, and 110 GPa for titanium plates, with a Poisson ratio of 0.3 for all. The plates and screws were scanned in 3-D by Smart Optics and saved as point clouds in stl format. The 3-D scans of the plates and the screws were then transferred to Rhinoceros software in stl format for the adaptation of the implants to the other sets. 

Models were converted into solid models as bricks and tetrahedral elements. In the bricks and tetrahedral modeling system, Fempro utilizes 8 noded elements provided that the 8 noded elements could reach the required detail levels. When 8 noded elements could not reach the required detail levels, instead of 8 noded elements, 7, 6, 5, or 4 noded elements were used sequentially. In the models employed in this study 921,517 to 1,329,954 elements were used. Models were regarded as homogeneous and isotropic materials entirely, to be proportional to the variability of deformation of the structure.

In constructed models, a load of 2.3 kN was applied from the upper surface of the sacrum at standing position and the displacements were analyzed by using a three-dimensional finite element stress analysis method. Motions by the fracture line were measured in x-, y-, and z-axes in four different points of interest (POIs) on both the pubic and iliac sides of the fracture line. Displacements of the fragments were calculated by subtracting the pubic side measurements from the iliac side. In the y-axis, positive values indicate compression stresses and negative values indicate displacement stresses at the fracture line. In x- and z-axes, positive values show medial and superior displacements, while negative values show lateral and inferior displacements, respectively. Additionally, loadings on the plate and screws were measured in standing positions. 

No statistical analysis can be done for any finite element analysis as the same motion and stress will be created at the same loading force and vectors, eliminating the probability.

## 3. Results

### 3.1. Displacement at the fracture line in infrapectineal plate models

The infrapectineal plate model with unlocked screws was found to be the most stable method in load-applied standing models when the motion of the fragments was evaluated by fracture line. The displacement values between the fragments were as low as 0 to 0.006 mm under loading in standing positions (Figure 2A). The infrapectineal model with combined screws had the second most stable fixation with 0 to 0.012 mm displacement in standing positions. The model with locked screws was still comparable with 0 to 0.017 mm displacements in various axes in standing positions (Table 1).

**Table 1:** Displacement at the fracture line in models in total and due to x-, y-, and z-axes.

Standing position				
	Pubic side		Iliac side	
	Screwlocation*	Highest maxstress (mPa)	Screwlocation*	Highest maxstress (mPa)
Infrapectineal				
Locked	5th hole	17.82	8th hole	71.87
Combined	5th hole	27.6	9th hole	392.72
Unlocked	5th hole	8.62	8th hole	41.4
Suprapectineal				
Locked	5th hole	24.72	9th hole	100.62
Combined	5th hole	454.25	9th hole	254.15
Unlocked	4th hole	681.37	8th hole	417.45

*Hole numbers are given from the most distal hole to most proximal one.

**Figure 2A:**
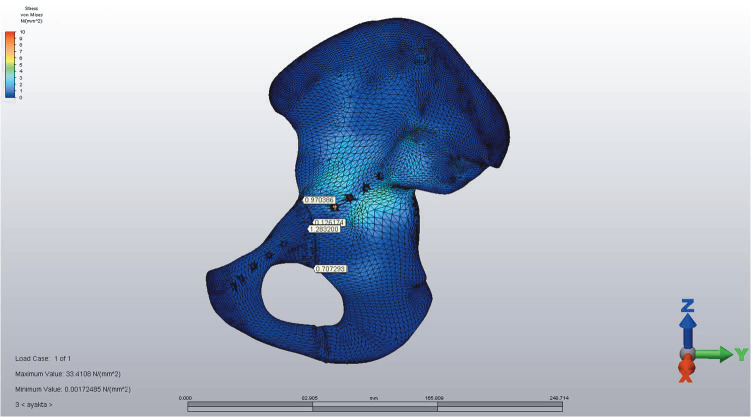
Infrapectineal model with unlocked screws. The displacement values between fragments were as low as 0 to 0.001 mm under loading in standing positions in all axes. Note that the plate is in close proximity to medial and lateral POIs and in balanced proximity to superior and inferior POIs.

**Figure 2B:**
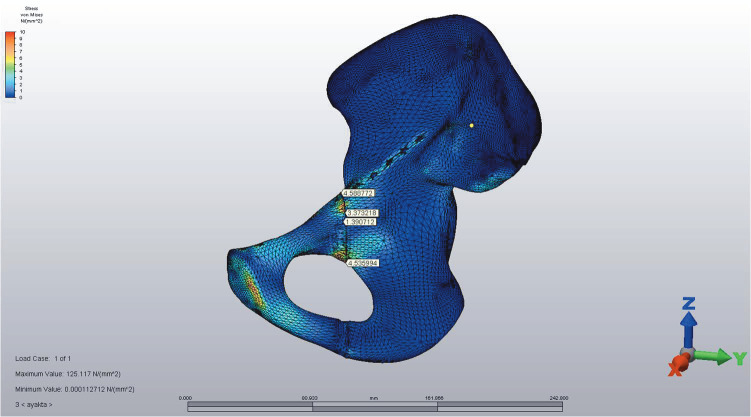
Suprapectineal plate model with unlocked screws was found to be the most unstable standing positions with 0.006 to 0.047 mm of displacement in various axes.

### 3.2. Displacement at the fracture line in suprapectineal plate models

The suprapectineal plate model with unlocked screws was found to be the most unstable in standing positions with 0.009 to 0.46 mm of displacement in various axes (Figure 2B). To be more precise, in standing position maximum displacement values were 0.46 mm vertical shear movement in the x-axis, –0.14 mm displacement in the y-axis, and –0.33 mm lateral shear in the z-axis (Table 1). The suprapectineal plate model with combined screws had 0.006 to 0.04 mm displacement in standing positions in various axes. The suprapectineal plate model with locked screws was the only model comparable to infrapectineal counterparts (particularly the locked infrapectineal model), with 0.006 to 0.034 mm displacement in standing positions in various axes (Table 1).

### 3.3. Maximum loads on plates

In standing position maximum stress on the plate was lowest in the infrapectineal model with unlocked screws with 25.3 mPa. In the suprapectineal unlocked model maximum stress on the plate was highest with 573.85 mPa (Table 2).

**Table 2:** Stress loading on plates.

Standing position				
	Highest maximumstress (mPa)	Stresslocation*	2nd highest maximumstress (mPa)	Stresslocation
Infrapectineal				
Locked	38.75	Between9th and 10th holes	26.85	Between8th and 9th holes
Combined	62.67	Between8th and 9th holes	26.45	Between5th and 6th holes
Unlocked	25.3	Between9th and 10th holes	22.42	Between8th and 9th holes
Suprapectineal				
Locked	41.8	Between9th and 10th holes	28.75	Between7th and 8th holes
Combined	152.95	Between2nd and 3rd holes	105.8	Between4th and 5th holes
Unlocked	573.85	Between2nd and 3rd holes	536.47	Between3rd and 4th holes

*Hole numbers are given from the most distal hole to most proximal one.

### 3.4. Maximum loads on screws

Among the maximum stress-bearing screws, the unlocked screws in the infrapectineal model had the lowest maximum stress loads with 41.4 mPa in standing positions. The suprapectineal model with unlocked screws had the highest maximum load with 681.37 mPa in standing positions (Table 3).

**Table 3:** Stress loading on screws.

Standing position				
	Pubic side		Iliac side	
	Screwlocation*	Highest maxstress (mPa)	Screwlocation*	Highest maxstress (mPa)
Infrapectineal				
Locked	5th hole	17.82	8th hole	71.87
Combined	5th hole	27.6	9th hole	392.72
Unlocked	5th hole	8.62	8th hole	41.4
Suprapectineal				
Locked	5th hole	24.72	9th hole	100.62
Combined	5th hole	454.25	9th hole	254.15
Unlocked	4th hole	681.37	8th hole	417.45

*Screw location numbers are given from the most distal hole to most proximal one.

## 4. Discussion

The aim of this study is to compare the stability and implant stresses of suprapectineal and infrapectineal plate models with subconfigurations for anterior column fractures. Acetabulum fractures can be fixed using a suprapectineal plate via an ilioinguinal approach (1,2) or using a infrapectineal plate (3,4) by a modified Stoppa approach. The modified Stoppa approach attracts attention as an important alternative to the ilioinguinal approach, having the advantages of permitting both suprapectineal and infrapectineal plate application under direct visualization of the quadrilateral surface with a relatively minimal invasive approach without the need for major vascular dissections. However, a comparison of the fixation stability of the conventional suprapectineal plates to infrapectineal plates has been researched only once. The recent study by Kacira et al. (11) indicated that infrapectineal and suprapectineal plates had no difference in stability against axial compression forces. However, the results of the current study clearly indicate that infrapectineal fixation models are much more stable and have much less implant stresses than the suprapectineal applications in standing positions. This finding is particularly important for experienced surgeons who are comfortable with both ilioinguinal and modified Stoppa approaches. By using infrapectineal plate fixation, the surgeon can be more comfortable in means of fracture stability. The superiority of the infrapectineal plates in terms of stability could be attributed to three factors. First, the infrapectineal plate supports the pelvic ring from the inner side, in which both ends of the plate form a sealed and more stable structure. Second, mechanically, it is easier to band a plate on the frontal aspect (2–3 mm) than on the side aspect (10 mm) due to differences of the inertia bending moment. As in standing positions the loads act vertically, the suprapectineal plate faces the loads frontally, whereas the infrapectineal plate faces loads on its side aspect. Third, if the locations of the plates are analyzed in reference to four POIs, in comparison to the suprapectineal plate, the infrapectineal plate could easily be detected in closer proximity to all POIs except the superior POI. Consequently, the infrapectineal plate supports four critical corners of the fractured acetabulum in a balanced manner, converting the standing loads to compression forces without evident shear. In accordance with our results, this might be more advantageous when unlocked screws are used, leaving some elasticity that would enable a degree of compression. Indeed, in our study, using unlocked screws in the infrapectineal model in standing positions provided a much more stable construction than using locked screws or using a combination of both screw types. However, it should be noted that locked screws may be more feasible in particularly osteoporotic patients, as conventional screws may not be durable enough to fix the fracture throughout the healing process. Some new anatomical plates include both suprapectineal and infrapectineal holes. While these designs can be helpful for stabilization of some specific fracture patterns, particularly those including posterior hemitransverse course or T-type fractures, we aimed to compare only the basic choices of fixation in a simple pattern of an anterior column fracture in this study. Future studies are needed to test these new-generation anatomical plates and also use both suprapectineal and infrapectineal plates orthogonally. 

The screw choice seems to also be important in application of conventional suprapectineal plates. The suprapectineal model subtype that employs entirely locked screws was more stable than the other suprapectineal models that use unlocked or combined screws. Similar to the infrapectineal plate model with combined screws, this subtype also had less stress loading on implants compared to other suprapectineal models. Our results suggest that this model could be preferred to supplement infrapectineal plates to fix unstable or osteoporotic fractures or in the case that infrapectineal fixation would not be possible due to factors related to the fracture or surgeon. On the other hand, the suprapectineal model with unlocked screws was the most unstable model with 22 to 49 times more motion than the infrapectineal model with unlocked screws. Furthermore, the highest maximum stresses in the plate and screws were detected in the suprapectineal model with unlocked screws. The suprapectineal model with unlocked screws had a maximum stress of 573.85 mPa on the plate and 681.37 mPa on the highest loaded screw, which is 23 to 29 times more for the plates and 17 to 21 times more for the screws that were used for the infrapectineal model with unlocked screws.

The pelvic model of the current study was an adaptation from another study on transverse acetabular fractures (14), with a significant difference. In the previous study a gap of 1 mm was left at the fracture line to better examine the compression forces. However, this gap does not allow any friction between the fragments, which might have been emphasized by compression of the fragments if the fragments were to be replaced in contact with each other. Thus, in the current study, fragments were left in contact and friction was allowed, contributing to the stability of the construct. Additionally, our model is superior to previous models with respect to the number of elements (921,517 vs. 1,329,954) that are used for modeling (14,15). Our model was extracted from a 39-year-old male cadaver from the Visible Human Project and the cortical to spongious bone ratio was 28/72, which is applicable for later young adulthood. 

Our study is limited by using computer programs rather than testing the mechanical elements in a real-world environment. However, the computer simulations have allowed us to better standardize the forces and the bony and implant structures for our experiments. Even though the strains and stresses on bone and implants in such a finite element analysis should not be directly and quantitatively interpreted to clinical practice, this method is very reliable in comparing two or more models of fracture fixation (16,17). Another limitation is the selection of an anterior column fracture model to represent the acetabular fractures where this type of fracture is observed occasionally among the acetabular fractures. However, this basic model with a single fracture line was preferred since this specific type can be treated either with suprapectineal or infrapectineal plate fixation in daily clinical practice. Besides, the calculations derived from this model can also provide insights into the biomechanics of pubic root fractures, in which the ideal screw locations are quite similar to those of the current model. Our results indicate that the suprapectineal plate fixation with unlocked screws, which is the most popular technique in clinical practice, is the least stable fixation configuration with the highest loading on the fixation materials among those tested in this study. On the contrary, infrapectineal plate fixation with unlocked screws seems to be the most reliable fixation type for anterior column fractures with the lowest displacement and least loading stresses on implants under loading. Accordingly, we recommend infrapectineal plate and unlocked screw fixation for similar fractures in clinical practice. When suprapectineal plate fixation is planned, the surgeon should consider using locked screws exclusively. In the future, clinical studies are needed to justify our findings in the finite element model. 

## Acknowledgments

The authors would like to thank TOTBİD for funding this study.
